# Comprehensive exercise recommendations for pediatric asthma: an evidence synthesis

**DOI:** 10.1007/s12519-025-00976-6

**Published:** 2025-10-11

**Authors:** Hong-Zhen Xu, Nan Lin, Guan-Nan Bai, Yu-Lin Liu, Xiao-Hui Jia, Cong Huang, Liang Hu, Han-Qing Shao, Qi-Yun Shang, Li-Fang Zhang, Ying-Shuo Wang, Yuan-Yuan Zhang, Lan-Fang Tang, Yun-Lian Zhou, Guo-Hong Zhu, Jian-Guo Hong, Zhi-Min Chen

**Affiliations:** 1 Nursing Department, National Clinical Research Center for Child Health, Children’s Hospital, Zhejiang University School of Medicine, Hangzhou, 310052 China; 2 Department of Child Health Care, National Clinical Research Center for Child Health, Children’s Hospital, Zhejiang University School of Medicine, Hangzhou, 310052 China; 3 Department of Respiratory, Children’s Hospital of Chongqing Medical University, Chongqing, 401122 China; 4 Department of Pediatric Respiratory Medicine, The Second Affiliated Hospital and Yuying Children’s Hospital of Wenzhou Medical University, Wenzhou, 325035 China; 5 Department of Sports Science, College of Education, Zhejiang University, Hangzhou, 310058 China; 6 Department of Pulmonology, National Clinical Research Center for Child Health, Children’s Hospital, Zhejiang University School of Medicine, Hangzhou, 310052 China; 7 Department of Pediatric Respiratory Disease, Anhui Provincial Children’s Hospital, Hefei, 230051 China; 8 Department of Respiratory and Critical Care Medicine, Kunming Children’s Hospital, Kunming, 650000 China; 9 Department of Pediatrics, Shanghai General Hospital Affiliated to Shanghai Jiaotong University School of Medicine, Shanghai, 200080 China

**Keywords:** Child, Asthma, Exercise, Exercise-induced bronchoconstriction, Recommendation

## Abstract

**Background:**

Bronchial asthma is a common chronic respiratory disease in children. For many years, concerns about exercise-induced bronchoconstriction have limited physical activity in this population, with negative consequences for both physical and mental health. Recent evidence indicates that exercise should be incorporated into the daily routine of children with asthma, with appropriately prescribed programs shown to improve disease control, lung function, and quality of life. This study aims to systematically describe the safety, benefits and key factors of exercise for children with asthma.

**Data sources:**

Initiated by the National Clinical Research Center for Child Health, this set of recommendations was developed by a multidisciplinary team of 17 experts. A comprehensive Literature search was conducted across PubMed, Embase, Cochrane and other databases, yielding 64 studies that met inclusion criteria up to May 2025. The Oxford Centre for Evidence-Based Medicine 2011 levels of evidence were used to evaluate evidence quality. Two rounds of expert voting were conducted using Delphi methodology to formulate final recommendations on key clinical topics.

**Results:**

Recommendations were formulated across nine core domains: exercise safety, exercise-related benefits, pre-exercise screening, exercise prescription design, plan adjustment and progression, pre-exercise preparation, exercise monitoring, outcome assessment and the management of exercise-induced bronchoconstriction. Specific guidance is offered on individualized exercise planning based on asthma control status, physical fitness, exercise habits and environmental factors. Recommendations also address appropriate modalities of aerobic, resistance and flexibility training, strategies for monitoring intensity and progression and both pharmacologic and non-pharmacologic approaches to exercise-induced bronchoconstriction prevention and management.

**Conclusions:**

These recommendations provide scientific and practical guidance for the development and implementation of individualized exercise prescriptions in children with asthma. Moreover, they highlight the importance of multidisciplinary collaboration and reinforce exercise as an integral component of asthma management. Further high-quality clinical research is needed to optimize exercise protocols and evaluate long-term outcomes.

**Graphical abstract:**

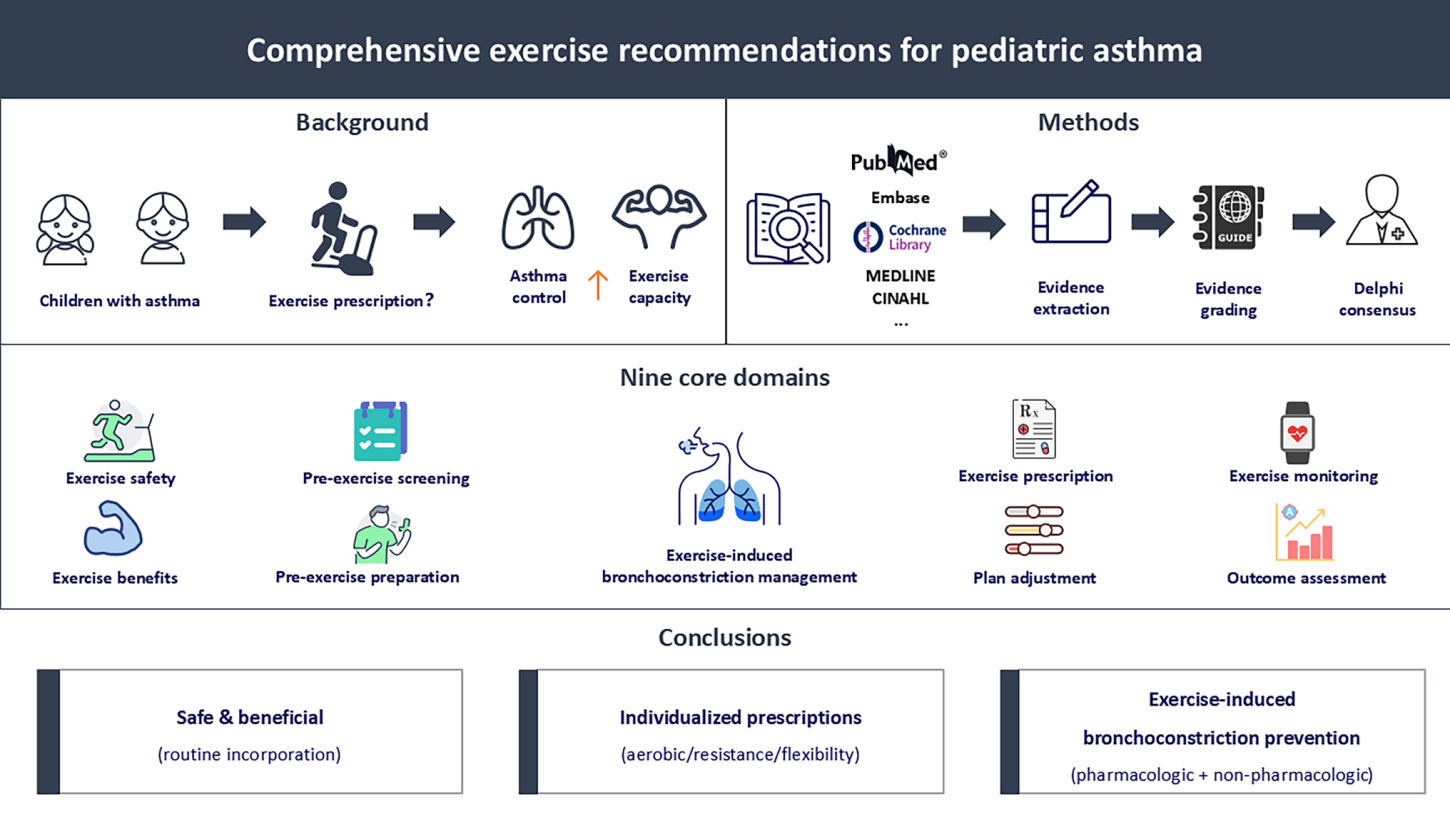

## Introduction

Bronchial asthma is the most common chronic respiratory allergic disease in childhood, with a global age-standardized prevalence of about 4757.84 per 100,000 children [1, 2]. The pathogenesis of asthma is complex and typically manifests as airway inflammation and airway hyperreactivity, leading to recurrent episodes of wheezing, breathlessness, chest tightness and coughing [3, 4]. Although asthma has become a significant public health issue for children worldwide, its management and treatment still face numerous challenges, especially when it comes to encouraging active participation in exercise for affected children.

For many years, parents have had concerns that physical exertion might trigger asthma attacks in their children. One key reason for this concern is exercise-induced bronchoconstriction (EIB), which refers to the exacerbation of asthma symptoms such as wheezing, coughing and shortness of breath, caused by airway constriction following physical exertion [5]. This belief has led to restrictions on physical activity for many children, potentially hindering their physical development and contributing to a range of health problems associated with a sedentary lifestyle, such as obesity [6]. However, with continuous advances in clinical research, an increasing body of evidence now shows that children with well-controlled asthma can safely engage in exercise. Moreover, exercise has a wide range of positive effects on the health of children with asthma, particularly in improving cardiopulmonary function, exercise tolerance and overall quality of life [7–9]. For instance, with proper pharmacological treatment and exercise interventions, lung function of many children with asthma has improved and exercise-induced asthma symptoms have been effectively controlled. Moreover, regular exercise also enhances the child’s mental health by reducing anxiety, depression and other psychological issues related to asthma [10]. These positive effects not only contribute to improving the physical health of children but also enhance their social interaction skills and emotional regulation.

However, the implementation of exercise must be individually designed according to the specific needs of each child with asthma. This includes considering their asthma control status, physical health, exercise habits and environmental factors. Clinical guidelines emphasize that exercise plans should be based on a thorough evaluation of symptoms, ensuring proper medication management before and after exercise. This includes using inhaled corticosteroids (ICS) and short-acting β_2_-agonists (SABA) to prevent EIB [11, 12]. In addition, environmental factors, such as air quality, are important considerations when designing exercise plans.

This article of recommendations aims to systematically describe the safety of exercise for children with asthma, the multiple health benefits of exercise and the key factors in developing exercise programs. It will also provide practical guidance on how to create personalized exercise plans for children with asthma, helping healthcare professionals and caregivers better integrate exercise into asthma management plans and improve the overall health of affected children.

## Methods

This study has been registered on the International Practice Guideline Registry Platform with the registration number PREPARE-2024CN1243.

### Working group

These recommendations were developed under the leadership of the National Clinical Research Center for Child Health and the Children’s Hospital, Zhejiang University School of Medicine. A multidisciplinary expert panel of 17 members was convened, including seven pediatric pulmonologists, seven respiratory nursing specialists, two sports medicine experts, and one epidemiologist. The panel comprised nine medical doctors, five individuals with master’s degrees, and three individuals with bachelor’s degrees, with experience ranging from 9 to 40 years in relevant fields. All experts had prior involvement in guideline development or relevant research. The panel members represented diverse regions and institutions across the country, ensuring broad regional representation.

### Data sources and search strategy

A comprehensive search was conducted across multiple databases, including PubMed, Embase, Cochrane Library, MEDLINE, CINAHL, Scopus, Web of Science and PsycINFO. The search spanned from the inception of these databases through May 2025 and was designed to identify publications relevant to the topics of “exercise” and “asthma” (Supplementary Table 1). In addition, reference lists and citations of identified relevant articles were manually reviewed to ensure comprehensive coverage. Authors of publications with incomplete or insufficient data were contacted to request the necessary additional information.

### Inclusion and exclusion criteria

Study inclusion criteria were as follows: the study population must consist of children or adolescents diagnosed with asthma; the study must involve physical exercise as a component; and document types eligible for inclusion were clinical guidelines, evidence summaries, best practice manuals, clinical decision support systems, systematic reviews, technical reports, clinical trials and quasi-experimental studies.

Studies were excluded based on the following criteria: duplicate publications, incomplete data, systematic reviews or meta-analyses already incorporated into existing guidelines, updated guidelines and study protocols of systematic reviews or meta-analyses. Original studies that were included in systematic reviews or meta-analyses and already incorporated into existing guidelines or evidence summaries were not considered as individual studies. A total of 13,835 studies were initially identified. After removing 6193 duplicates, 7642 studies remained for screening. Following abstract and title review, 7526 studies were excluded. After full-text review, 21 studies were excluded as they focused on adults, 13 for non-exercise respiratory training, nine for animal studies and eight for irrelevant research types. Finally, 64 studies were included in the review.

### Evidence grading

Evidence retrieved from the selected studies was assessed using the Oxford Centre for Evidence-Based Medicine 2011 Levels of Evidence (OCEBM) (Supplementary Table 1) [13]. Two researchers, trained in evidence appraisal using OCEBM criteria, independently evaluated the evidence (Kappa = 0.85). In case of disagreement, the evidence was discussed with a third methodologist (BGN) until agreement was reached.

### Process of reaching consensus

A Delphi method was applied to reach consensus on topics where information was either insufficient or contradictory. This is a reliable and validated technique used to obtain expert agreement. A two-round series of statement drafts was circulated via email for voting. Before the first round of Delphi voting and based on a literature review, researchers proposed eight main clinical topics. In the first round, expert panel members were invited to score the importance and feasibility of each topic using a five-point Likert scale. Experts were encouraged to comment on each issue and suggest new questions. Any issue that received an average score above 3.5 and a coefficient of variation below 0.30 was retained. Based on comments from these experts, some issues were modified, added, or removed. As a result, one clinical issue (Exercise safety) was added to the second round. In the final round of online voting, experts provided recommendations for each clinical issue. Voting options included “agree”, “disagree” and “uncertain”. A recommendation was considered reached when more than two-thirds of the expert panel members chose “agree” (Table 1).

**Table 1:** Summary of the general evidence relevant to the exercise prescription

Domains	Evidence statement	Levels of evidence
Exercise safety	For most children with asthma, exercise is safe when their asthma is well-controlled through appropriate pharmacological treatment, and individualized exercise plans are implemented to ensure safety while optimizing health benefits	Level 1
Exercise benefits		
Asthma management	Exercise improves asthma control, reduces the frequency of acute exacerbations, decreases the need for medication, alleviates nocturnal symptoms, and enhances sleep quality	Level 1
Cardiopulmonary function	Aerobic exercise enhances cardiorespiratory fitness and mitigates the risk of EIB. Specific activities, such as swimming, have been shown to improve pulmonary function in children with asthma	Level 1
Mental and social well-being	Exercise contributes positively to mental health, quality of life, and social skills in children with asthma	Level 1
Preexercise screening		
Asthma control	Well-controlled: exercise should align with the general guidelines for healthy children	Level 2
	Partially controlled: begin with low-intensity, short-duration exercise, gradually increasing intensity and duration as tolerated	Level 2
	Uncontrolled: only well-tolerated physical activities should be undertaken until asthma control improves	Level 1
Exercise habits^a^	For individuals without a regular exercise habit: initiate with low to moderate-intensity exercise, progressively increasing intensity	Level 5
	For individuals with a regular exercise habit: maintain moderate to high-intensity exercise	Level 5
Cardiopulmonary function	Cardiopulmonary function can be assessed through maximal or submaximal exercise testing. Key parameters to monitor during testing include heart rate, blood pressure, respiratory rate, perceived breathlessness, oxygen saturation, and subjective fatigue	Level 1
Body composition	Measure height and weight to calculate BMI-Z for assessing nutritional status	Level 1
	For children with suboptimal nutritional status, low muscle mass, or poor motor stability, avoid exercises requiring high intensity or explosive power to minimize injury risk	Level 5
Age	Preschool children: focus on developing basic motor skills primarily through play	Level 1
	School-aged children: avoid excessive high-intensity exercise and weight-bearing resistance training. Suitable body-weight resistance exercises can be performed	Level 4
Environment	The ideal temperature for exercise in children with asthma is between 20 and 24 °C	Level 3
	Relative humidity should be above 40% or absolute Humidity should exceed 10 g/m^3^	Level 2
	PM2.5 concentrations should be below 10 μg/m^3^, and exposure to allergens should be avoided	Level 2
Exercise promotion & barriers	Evaluate the child’s family, school, and social resources, as well as available discretionary time, to optimize the exercise plan	Level 5
	Use behavioral theories to identify factors influencing exercise participation, aiding professionals in developing strategies to promote and sustain regular exercise	Level 5
Exercise plan development		
Aerobic exercise	Frequency: 3 to 5 times/wk	Level 1
	Intensity: begin with moderate intensity, progressively increasing. For sedentary children or those with low fitness levels, initiate with low to moderate intensity	Level 2
	Time: progressive to 60 min of moderate-to-vigorous-intensity exercise per day	Level 2
	Type: include a variety of exercises involving large muscle groups, such as walking, jogging, swimming, cycling, ball sports, and intermittent exercise	Level 1
Resistance training exercise	Frequency: 2–3 times/wk, with at least 48 h between sessions targeting the same muscle group	Level 1
	Intensity: recommended intensity is 60%–70% of 1-RM. For sedentary individuals or those with lower fitness levels, start at 40%–50% of 1-RM	Level 2
	Time: perform 2–4 sets per muscle group, with 8–12 repetitions per set and 2–3 min of rest between sets	Level 2
	Type: target all major muscle groups, prioritizing multi-joint bodyweight exercises (e.g., push-ups, pull-ups, squats, crunches). Resistance exercises using equipment, such as dumbbells, can also be incorporated	Level 2
Flexibility exercise	Frequency: 2–3 times/wk	Level 1
	Intensity: stretch to the point of mild tightness or discomfort	Level 2
	Time: repeat each stretch 2–4 times, holding for 10–30 s, with a total duration of 60 s	Level 4
	Type: include static stretching (e.g., chest stretch, side leg press, knee joint exercises, wrist and ankle joint exercises) and dynamic stretching (e.g., stationary short-distance jogging at low-to-moderate intensity)	Level 5
Plan adjustment & progression		
Follow-up & adjustment	The first follow-up should occur 1–2 wk after implementation, with subsequent follow-ups every 4 wk to progressively optimize exercise content and intensity	Level 5
Progressive plan	In the first 4–6 wk, gradually increase training duration by 5–10 min every 1–2 wk. After 1 mon of regular exercise, progressively adjust duration, frequency, and/or intensity over the next 4–8 mon, reaching the recommended exercise volume and quality	Level 5
Special periods	During respiratory infections or exposure to allergens (e.g., flu season, pollen season, poor air quality), increase follow-up frequency. Adjust exercise type, intensity, frequency, and duration under specialist guidance to ensure safety and address individual needs	Level 5
Pre-exercise preparation		
Lung function monitoring	Use a handheld home spirometry device prior to exercise to obtain lung function data, enabling more effective monitoring of airflow limitation and facilitating personalized exercise recommendations by healthcare professionals	Level 2
Medication preparation	Carry a SABA during exercise	Level 2
	For children with a history of EIB, preventive medication should be administered 10–20 min before exercise	Level 2
Warm-up	Warm-up for about 10 min with low-to-moderate intensity activities to help prevent EIB and improve exercise tolerance	Level 4
Exercise equipment	Prepare appropriate gear, including clothing, shoes, equipment, and a water bottle. In cold environments, use a mask to warm and humidify inhaled air	Level 5
Exercise monitoring		
Intensity assessment	Subjective assessment: e.g., the RPE scale and the talk test. Relative measures: e.g., HRR, VO_2_R, maximum heart rate, and maximum oxygen uptake methods. Absolute measures: e.g., the METs method	Level 2
Real-time monitoring	Use wearable fitness trackers (e.g., fitness bands or smartwatches) for real-time monitoring of health data, including heart rate, respiratory rate, oxygen saturation, steps, and activity level during exercise	Level 2
Effectiveness assessment		
Physical fitness	Assess the child’s nutritional and physical development by measuring height, weight, and calculating BMI-Z	Level 2
Asthma control	Regularly use spirometry to assess the impact of exercise on asthma control. When utilizing asthma control questionnaires, longitudinal tracking of scores is recommended to provide more reliable insights into symptom perception over time	Level 2
Cardiopulmonary function	Assess cardiopulmonary function through maximal or submaximal exercise testing	Level 2
Quality of life and psychological health	Assess improvements in the child’s psychological state and well-being using asthma-specific quality of life questionnaires and anxiety/depression scales	Level 1
EIB management		
EIB identification	EIB should be diagnosed based on characteristic symptoms, such as cough, wheezing, dyspnea, and chest tightness, occurring within 15 min after 5–8 min of intense exercise. Diagnosis should be confirmed with exercise challenge testing and spirometry, demonstrating a ≥ 10% decrease in FEV1. Spirometry should be performed as soon as possible after exercise, particularly in younger children	Level 1
Pharmacological treatment	SABAs should be used 5–20 min before exercise. If symptoms persist or SABA use is frequent (daily or more), daily ICS or LTRAs are recommended for long-term control	Level 1
Non-pharmacological interventions	Avoid cold, dry, or polluted environments, and perform warm-up exercises	Level 2
Lifestyle modifications	Regular exercise, weight control, and maintaining overall physical fitness are recommended to reduce EIB occurence	Level 5

^a^A regular exercise habit refers to engaging in planned, systematic physical activity of moderate intensity for at least 30 minutes per session, at least three days per week, sustained for a minimum of three months

*BMI-Z* body mass index for age, *EIB* exercise-induced bronchoconstriction, *FEV*_*1*_ forced expiratory volume in one second, *ICS* inhaled corticosteroids, *LTRA* leukotriene receptor antagonists, *METs* metabolic equivalents, *HRR* heart rate reserve, *RPE* rating of perceived exertion, *SABA* short-acting β2-agonists, *VO*_*2*_*R* oxygen uptake reserve, *1-RM* one-repetition maximum

## Results

### Question 1: Is exercise safe for children with asthma?

Recommendation: Exercise should be incorporated into the daily routine of children with asthma, with restrictions considered only in cases of severe EIB.

Evidence summary: Although concerns about EIB have historically led many parents to limit physical activity for children with asthma, accumulating evidence indicates that, for the majority of children, moderate exercise is both safe and beneficial [14–18]. Research indicates that many children with asthma tolerate physical activity well when supported by effective pharmacological management and individualized exercise plans [19, 20]. Optimal asthma control and appropriate medication use are essential to prevent exercise-induced airway constriction. Clinical guidelines emphasize the need for thorough assessment and monitoring of symptoms and lung function before, during and after exercise to ensure safety [21]. Before exercise, healthcare providers should evaluate symptoms and pulmonary status, confirm the use of preventive medications (e.g., ICS and SABA) and assess exercise suitability [22]. Hengeveld et al. [23] proposed incorporating exercise challenge tests (ECT) into the evaluation of respiratory symptoms, as this method helps assess both exercise capabilities and respiratory symptoms, ensuring the child can safely engage in exercise. During exercise, especially in children prone to EIB, monitoring respiratory rate, heart rate and airway symptoms (e.g., coughing, wheezing) is critical [24, 25]. Post-exercise, symptom monitoring and pulmonary function tests [e.g., forced expiratory volume in one second (FEV_1_)] may be required to assess airway response. In conclusion, most children with asthma can safely engage in physical activity with proper asthma management. Personalized exercise programs and regular health assessments are critical for optimizing safety and maximizing exercise benefits.

### Question 2: What are the health benefits of exercise for children with asthma?

Recommendations: (1) Asthma management: exercise improves asthma control, reduces the frequency of acute exacerbations, decreases the need for medication, alleviates nocturnal symptoms and enhances sleep quality. (2) Cardiopulmonary function: aerobic exercise enhances cardiopulmonary function and reduces the risk of EIB. (3) Mental and social well-being: regular exercise positively affects mental health, quality of life and social skills, contributing to improved emotional regulation, reduced anxiety and depression and better social interactions.

Evidence summary: Growing evidence supports the beneficial role of exercise in asthma management. Regular exercise has been shown to improve symptom control [26–28], reduce medication use [29] and decrease the frequency of emergency visits and hospitalizations in children with asthma [14, 30]. A systematic review examining the impact of exercise on nocturnal asthma symptoms reveals that regular physical activity can substantially alleviate night-time symptoms, thus improving sleep quality [31]. However, while the positive effects of exercise on symptom control are well-established, its role in modulating airway inflammation and enhancing immune function remains unclear [32]. Some studies suggest that exercise may reduce airway inflammation, as indicated by lower fractional exhaled nitric oxide (FeNO) levels [33]; more research is needed to clarify the mechanisms involved.

Aerobic exercise improves cardiorespiratory function and exercise capacity in children with asthma, including an increase in maximal oxygen uptake [26, 30, 33–36]. Although current evidence does not provide strong support for the notion that general exercise interventions directly improve lung function, studies have indicated that specific types of exercise, such as swimming, may help improve lung function and reduce EIB symptoms [37, 38]. The rhythmic nature of swimming and the exposure to moist air may further reduce respiratory tract irritation, providing additional benefits for children with asthma.

Exercise has been shown to positively impact the psychological health and overall quality of life of children with asthma. A significant body of evidence consistently indicates that regular physical activity improves the quality of life of children with asthma [10, 18, 26, 39, 40]. Specifically, children who participate in regular exercise experience reductions in the symptoms of anxiety and depression that are associated with asthma [36]. Psychological benefits of exercise are most evident as improvements in emotional regulation and social interaction skills; these are vital for overall health and well-being [41].

### Question 3: What key factors should be considered when formulating an exercise plan for asthmatic children?

#### Question 3a: How does asthma control influence the formulation of an exercise plan?

Recommendations: For children with well-controlled asthma, exercise should align with the general guidelines for healthy children. For children with partially controlled asthma, initiation of low-intensity, short-duration exercise, with gradual increases in intensity and duration as tolerated is recommended. For children with poorly controlled asthma, only tolerable physical activity should be undertaken until asthma control improves.

Evidence summary: Asthma control is essential to determine exercise safety. This is usually assessed using pulmonary function tests and validated questionnaires, such as the childhood asthma control test (C-ACT), asthma control test (ACT) and test for respiratory and asthma control in kids (TRACK), which are commonly used in clinical practice and research [42]. Peak expiratory flow (PEF) monitoring is another useful tool for home-based assessment, tracking asthma control via PEF values and their variability [43]. However, questionnaires may be unreliable in children and PEF measurements are prone to manipulation, which may not provide sufficient insight into expiratory flow limitation. Advances in technology have introduced portable spirometers and smartphone apps, allowing children to perform spirometry tests at home. Although not directly comparable to laboratory results, these tools provide better insights into expiratory flow limitation and allow professionals to assess measurement quality through flow-volume loops. Healthcare providers must identify children at risk of asthma deterioration and tailor exercise plans accordingly. For high-risk children, exercise should be restricted, while others, particularly those with partially controlled asthma, can engage in exercise if symptoms are controlled, with SABA available as needed. Several studies have shown that children with well-controlled asthma can safely exercise, while those with poorly controlled asthma are at higher risk for exacerbations or EIB [40, 44]. Clinical guidelines recommend that exercise be postponed for children with acute asthma exacerbations or poor control until their condition stabilizes [21, 45–47]. Exercise intervention studies typically exclude children with recent flare-ups or medication changes to avoid worsening symptoms. Most randomized controlled trials (RCTs) focus on children with mild-to-moderate asthma, with limited data available on those with severe asthma. Studies, including that of Fanelli [40], show that while children with severe asthma are more likely to experience EIB, appropriate exercise interventions can still positively impact asthma management. Even with the potential for EIB, exercise can reduce the incidence of EIB and alleviate exercise-induced dyspnea, suggesting long-term benefits for asthma control.

#### Question 3b: How should exercise habits be considered when designing an exercise plan?

Recommendations: Children without a regular exercise habit should start with low- to moderate-intensity exercise, gradually increasing in intensity. For children who already have a regular exercise routine, moderate to high-intensity exercise should be maintained.

Evidence summary: Assessing exercise habits is essential for developing a personalized exercise plan for children with asthma. The American College of Sports Medicine (ACSM) recommends conducting an exercise history screening to assess an individual’s physical activity regularity, tolerance and potential risks [48]. According to ACSM, individuals who engage in moderate-intensity exercise at least three times a week for at least 30 minutes per session, sustained for more than three months, can be considered “regular exercisers” [48]. This screening helps identify children who have not yet adapted to regular exercise, as they may be at higher risk of overexertion and injury. It is crucial to tailor exercise intensity based on the exercise history and cardiopulmonary function of the child to ensure safety and effectiveness.

#### Question 3c: How should cardiopulmonary function be assessed to ensure safe participation in exercise?

Recommendations: Cardiopulmonary function can be assessed using maximal or submaximal exercise testing. Key parameters to monitor during testing include heart rate, blood pressure, respiratory rate, perceived breathlessness, oxygen saturation and perceived exertion.

Evidence summary: Cardiopulmonary exercise testing (CPET) is considered the “gold standard” for assessing cardiorespiratory fitness. It measures maximum oxygen uptake (VO_2max_) and provides detailed physiological data on metabolic parameters and exercise capacity [49, 50]. However, due to its complexity, high cost and the requirement for maximal exertion, CPET may not be necessary in most cases. An alternative approach, the ECT as recommended by the European Respiratory Society (ERS) and American Thoracic Society (ATS), provides a viable alternative. This test, typically performed using a treadmill or cycle ergometer, effectively evaluates exercise tolerance and cardiorespiratory function without necessitating maximal intensity or expensive equipment. The six-minute walk test (6MWT) is another commonly used submaximal test, especially for children with low fitness levels or chronic respiratory conditions [51, 52]. Standardized by the ATS, the 6MWT protocol makes a reliable and clinically valuable tool for assessing exercise tolerance and guiding exercise prescriptions [53].

During exercise testing, key physiological parameters, such as heart rate (HR), blood pressure (BP), respiratory rate, perceived breathlessness, oxygen saturation and subjective fatigue, should be monitored to ensure safety and a reliable assessment [48]. HR should be measured at the 2nd and 3rd minutes of the test to assess cardiac load and recovery trends. BP should be recorded one minute before the test ends to assess hemodynamic responses. Respiratory rate and oxygen saturation should be monitored continuously throughout the test. Perceived exertion can be assessed using the Borg rating of perceived exertion (RPE) scale, and perceived breathlessness can be evaluated using a visual analog scale or similar method, both during the final minute of exercise to assess the participant’s perception of exercise intensity and comfort. These parameters help in making necessary adjustments to exercise intensity and in evaluating potential risks during exercise.

#### Question 3d: How should body composition be assessed when creating an exercise plan?

Recommendations: Children’s height and weight should be measured to calculate their body mass index (BMI)-Z score for assessment of nutritional and developmental status. For children with suboptimal nutritional status or low muscle mass, exercises requiring high intensity or explosive power should be avoided to minimize the risk of injury.

Evidence summary: Nutritional status and body composition directly affect exercise capacity and risks. Obese children may have more severe asthma symptoms and lower exercise tolerance due to increased fat mass, reduced lung function and chronic airway inflammation [6]. Malnourished children or those with low muscle mass are at greater risk for falls and injuries during exercise [54]. Measuring height, weight, and calculating BMI-Z scores is recommended before intervention to assess nutritional status and identify malnutrition or growth deviations [55].

Obese children often have poor motor coordination and insufficient core strength, requiring caution during high-intensity exercises [56, 57]. Children with low muscle mass should avoid explosive strength exercises to minimize injury risk [58]. In addition, obese children may experience discomfort during high-intensity activities, leading to lower exercise compliance [59, 60]. Personalized interventions, such as gamified exercises, have been shown to improve long-term adherence [61, 62]. For malnourished children, assessing motor control and stability is crucial before starting exercise. A gradual, low-intensity approach, combined with nutritional support, enhances physical development and tolerance to exercise. While BMI-Z is a valuable tool, it does not distinguish between muscle and fat mass, and combining it with other measurements like skinfold thickness or handgrip strength can improve accuracy.

#### Question 3e: How do age-related factors influence exercise plan?

Recommendations: For preschool children, the focus should be on developing basic motor skills primarily through play. For school-aged children, it is recommended that excessive high-intensity exercise and weight-bearing resistance training is avoided to ensure safe physical development.

Evidence summary: Children at different ages have distinct physical, motor, and psychological characteristics, which influence their exercise needs and adaptability. For preschool-aged children, the focus should be on developing basic motor skills such as walking, running, jumping, throwing and balancing [63]. The primary goal at this stage is to enhance these abilities through playful, unstructured activities, with guidelines emphasizing free play and parent–child interactions rather than structured or competitive training [64–66].

In school-aged children, physical abilities are improving, but bones, tendons and joints are still developing, limiting their tolerance for impact and load. Excessive high-intensity exercise or heavy resistance training should be avoided to reduce the risk of musculoskeletal injuries, especially exercises involving large, uncontrolled movements or explosive force [67]. Safer alternatives include bodyweight resistance training, basic coordination exercises and fun group activities. While structured exercise plans can be beneficial for children with specific needs or conditions, for most healthy children, it is essential to prioritize unstructured, natural play for physical and motor skill development. Encouraging free play, physical exploration and non-competitive activities fosters better engagement and supports overall development.

Current research on exercise interventions for those with asthma primarily focuses on adolescents and adults, with limited studies on preschool and prepubertal children. These studies often have small sample sizes with short follow-up periods, which limits their long-term applicability. In addition, cultural differences and variations in educational systems may influence exercise patterns and affect the generalizability of intervention outcomes.

#### Question 3f: What environmental factors should be considered when planning exercise?

Recommendations: The ideal exercise environment for children with asthma should be at a temperature of 20–24 °C, with either a relative Humidity above 40% or an absolute Humidity above 10 g/m^3^. Concentrations of fine particulate matter less than 2.5 μm in diameter (PM2.5) should be below 10 μg/m^3^, and allergen exposure should be avoided.

Evidence summary: Both low and high temperatures can exacerbate the occurrence of EIB. Cold air cools the airways, leading to airway constriction and worsening symptoms, while hot air can dry out the airways, triggering similar respiratory reactions. Therefore, a temperature range of 20–24 °C is considered optimal from EIB-related experiments. This range helps avoid the negative impact of extreme temperatures on the airways, controls the cooling effect, and reduces the occurrence of EIB [68, 69]. In addition, humidity is another critical factor influencing EIB. The study of Tikkakoski indicates that when the absolute humidity (AH) is below 5 g/m^3^, the incidence of EIB significantly increases, whereas when the AH reaches or exceeds 10 g/m^3^, the risk of EIB is lower [70–72]. Furthermore, the study of Stensrud suggests that when relative humidity (RH) is Maintained above 40%, the incidence of EIB is reduced and further decreases when humidity exceeds this threshold [73]. However, current studies have not established a precise upper limit for humidity, with many simply confirming that higher humidity is generally associated with lower EIB incidence.

Air quality is a central factor in exercise safety [74]. According to guidelines, outdoor activities are generally considered safe when the air quality index (AQI) is between 0 and 100; this indicates good air quality, which is ideal for exercise [75, 76]. However, when the AQI is between 101 and 200 (moderate pollution), the intensity and duration of outdoor activities should be Limited, or less polluted times and areas should be chosen for low-intensity exercise. If the AQI exceeds 200 (severe pollution), outdoor exercise should be avoided to reduce exposure to harmful irritants. The negative impact of air pollution on asthma is well-established and outdoor exercise on polluted days can exacerbate symptoms. Asthmatic children should avoid outdoor activities during high-pollution days, pollen peaks, or when the weather is cold and dry. Notably, studies have shown that PM2.5 concentrations above 10 μg/m^3^ are associated with an increased incidence of EIB in children with asthma [70, 77]. If outdoor exercise is necessary, protective measures such as wearing masks to warm and humidify the air can help minimize exposure to harmful pollutants [45]. Although longer durations of outdoor activity have been shown to be protective against asthma, this benefit may be offset under poor air quality or in the presence of other environmental risk factors [78]. While allergen exposure, such as pollen, may be difficult to avoid entirely, it should not prevent children from exercising. In these cases, alternatives such as indoor exercise or choosing areas with lower pollen levels can help minimize exposure. In addition, using medications like antihistamines can effectively manage symptoms, allowing children with asthma to safely continue physical activity.

In addition to these outdoor and allergen-related considerations, indoor exercise environments also present potential risks. Poorly ventilated indoor spaces may contain asthma triggers such as volatile organic compounds (VOCs), mold and dust, which can exacerbate respiratory symptoms [79–81]. Swimming, often recommended for asthmatic children due to the warm and humid environment that helps reduce airway irritation, is generally considered safe. However, in indoor pools with inadequate ventilation, chloramine gases may accumulate and irritate the airways. Ensuring proper ventilation is, therefore, essential when engaging in indoor swimming activities.

#### Question 3g: What strategies can promote exercise participation and overcome barriers?

Recommendations: A thorough evaluation of family, school and social resources should be conducted to optimize the exercise plan for the child. Behavioral theories should be used to identify factors influencing exercise participation, helping professionals to develop strategies that promote and sustain regular physical activity.

Evidence summary: Children with asthma can reap significant health benefits from regular physical activity, but participation and adherence are often hindered by various factors. Identifying and addressing these barriers is essential for developing effective, sustainable exercise plans. Research highlights that family, school and societal resources are crucial external factors influencing exercise adherence in children with asthma [82, 83].

At the family level, studies indicate that parental exercise habits, attitudes and support play a significant role in a child’s physical activity levels. Activities such as parent–child exercise and encouragement are particularly effective [84, 85]. Parents also serve as key role models, with a positive correlation between parental and child activity levels, especially for moderate-to-vigorous-intensity exercise [86]. Creating a family-oriented exercise environment is essential, particularly for preschool-aged children.

At school, flexible physical education curricula, understanding of asthma by teachers, and their ability to manage emergencies are important to ensure children can participate safely [87, 88]. Social factors, including access to community exercise spaces, air quality and medical support, also affect opportunities for physical activity. High air pollution and limited exercise facilities significantly constrain exercise options [89].

Integrating exercise plans into asthma action plans is recommended to improve adherence, providing parents and children with a sense of safety and trust in managing asthma [21, 35]. Setting clear, measurable goals and tracking progress further motivates children. Motivational interviewing (MI) has also been shown to effectively improve exercise participation, especially for children at the preparation or hesitation stage [90, 91].

Theoretical models, such as self-determination theory (SDT) [92], theory of planned behavior (TPB) [93], social cognitive theory (SCT) [94] and the transtheoretical model (TTM) [21, 95], provide frameworks for understanding and predicting exercise behavior. These models help guide the design of interventions that foster intrinsic motivation, autonomy, role modeling and behavioral change at different stages of readiness.

### Question 4: What are the key recommendations for aerobic exercise?

Recommendations: (1) frequency: 3–5 times per week; (2) intensity: start with moderate intensity, gradually increasing. For sedentary children or those with low fitness, begin with low to moderate intensity; (3) time: progressive to 60 minutes of moderate-to-vigorous-intensity exercise per day; (4) type: include a variety of exercises involving large muscle groups, such as walking, jogging, swimming, cycling, ball sports and intermittent exercise.

Evidence summary: Extensive research supports that systematic aerobic training enhances pulmonary function, cardiorespiratory adaptation and quality of life for children with asthma [10, 30, 39, 96].

Regarding frequency, the World Health Organization (WHO) recommends at least three days of moderate to vigorous physical activity per week [66, 97]. Two systematic reviews suggest three to five sessions per week to effectively improve exercise capacity and cardiorespiratory function [10, 18]. For children with poorly controlled symptoms, reducing intensity and increasing frequency may be beneficial to manage the load better [48]. For intensity, most studies advise starting with moderate intensity, progressively increasing over time [10, 98]. Moderate intensity is defined as 40%–60% of maximal oxygen uptake reserve and has been shown to effectively improve cardiorespiratory function [45, 48]. Caution is needed, as prolonged or high-intensity exercise may trigger EIB, especially for those with poor fitness or uncontrolled symptoms. It is recommended that children engage in 60 minutes of moderate-to-vigorous physical activity (MVPA) daily, consistent with current pediatric guidelines. For children with asthma, total duration can be cumulative throughout the day via multiple shorter bouts of activity (“exercise snacks”). This can improve adherence and accommodate individual tolerance and asthma control status [45, 48, 66]. For type, exercises that engage large muscle groups, such as walking, jogging, cycling, swimming and team sports, are recommended [48]. Avoid strenuous outdoor activities in poor air quality or extreme temperatures and opt for non-chlorinated or well-ventilated swimming pools [45].

Recently, the value of interval training (IT) for children with asthma has garnered increasing attention. This training mode alternates between short bursts of high-to-maximal intensity exercise (45–240 seconds) and longer recovery periods of moderate-to-low intensity (60–360 seconds), thereby increasing overall training volume and average intensity [48]. Systematic reviews and meta-analyses show that short-term (≤ 3 months) high-intensity interval training (HIIT) is as effective as traditional continuous aerobic exercise in improving cardiorespiratory function, asthma symptom control and quality of life, with some indicators even showing superior results [8, 44]. IT distribution of effort makes it easier for children to adhere to the regimen, especially those with shorter attention spans [99]. However, current research on IT has limitations, including small sample sizes, inconsistent intervention protocols and unclear age stratification. Differences in parameters such as “maximum intensity” and “interval duration” across studies highlight the need for standardized, widely applicable clinical pathways.

### Question 5: What are the key recommendations for resistance training exercise?

Recommendations: (1) frequency: 2–3 times per week, with at least 48 hours between sessions targeting the same muscle group; (2) intensity: recommended intensity is 60%–70% of one-repetition maximum (1-RM). For sedentary children or those with lower fitness levels, start at 40%–50% of 1-RM; (3) time: perform 2–4 sets per muscle group, with 8–12 repetitions per set and 2–3 minutes of rest between sets; (4) type: target all major muscle groups, prioritizing multi-joint bodyweight exercises (e.g., push-ups, pull-ups, squats and crunches). Resistance exercises using equipment, such as dumbbells, can also be incorporated.

Evidence summary: Children with asthma undergoing long-term corticosteroid treatment often face side effects such as muscle atrophy and decreased strength, with those suffering from severe asthma particularly prone to lower muscle mass [100]. Regular resistance training can significantly improve limb strength and body composition in these children, potentially mitigating muscle degradation caused by steroid use, especially in those with lower fitness levels or those on long-term medication management [35]. Therefore, developing individualized, progressive resistance training programs once their condition is stable is of considerable clinical importance.

According to the ACSM and Australian Physical Activity Guidelines, children and adolescents should engage in 2–3 resistance training sessions per week, allowing at least 48 h between sessions for muscle recovery [45, 48]. Resistance training is often combined with aerobic exercises in these programs. A 12-week RCT involving children with mild-to-moderate asthma by Sanz-Santiago et al. [35] showed that combining three sessions of 60 minutes of both aerobic and resistance training per week resulted in significant improvements in cardiopulmonary health and muscle strength, with no severe adverse events reported. This frequency aligns with the WHO recommendations for strength training in children and adolescents [101].

Training intensity is often expressed as a percentage of the 1-RM. This is defined as the maximum weight an individual can lift for one repetition of a given exercise with proper form, without compromising safety [48]. Most studies suggest using 60%–70% of 1-RM as an effective range for improving strength in most asthmatic children with normal fitness levels [48]. For those with lower fitness levels or more sedentary behavior, starting at 40%–50% of 1-RM is recommended to minimize the risk of EIB or breathlessness.

Training protocols generally involve performing 2–4 sets of 8–12 repetitions per Major muscle group, with 2–3 minutes of rest between sets [48]. Exercises should target all major muscle groups, with a focus on compound multi-joint movements such as squats, push-ups, pull-ups and sit-ups. For younger or less fit children, resistance bands or light dumbbells can be incorporated to maintain interest and provide control over intensity.

### Question 6: What are the key recommendations for flexibility exercise?

Recommendations: (1) frequency: 2–3 times per week; (2) intensity: stretch to the point of mild tightness or discomfort, but not pain; (3) time: repeat each stretch 2–4 times, holding for 10–30 seconds, with a total duration of 60 seconds per stretch; (4) type: include both static and dynamic stretching exercises. Static stretches can include chest stretches, side leg presses, knee joint exercises, wrist and ankle joint exercises. Dynamic stretches might involve activities like stationary short-distance jogging at low-to-moderate intensity.

Evidence summary: Flexibility training plays a key role in improving muscle and joint range of motion, preventing injuries and enhancing physical function. While research on flexibility training for children with asthma is limited, evidence supports its positive impact on pulmonary rehabilitation. Flexibility exercises can improve joint mobility, respiratory muscle compliance and overall exercise performance [102].

Flexibility training is typically divided into static and dynamic stretches. Static stretches, such as chest expansion, lunges and ankle rotations, are primarily used for cooling down and muscle relaxation after exercise. Dynamic stretches, such as arm swings and dynamic lunges, are used for warming up to increase body temperature, improve neuromuscular excitability and reduce injury risk during physical activity [103]. Guidelines recommend that children engage in flexibility training 2–3 times per week, incorporating both static and dynamic stretching. Static stretches should be held for 10–30 seconds, repeated 2–4 times per stretch, totaling around 60 seconds per stretch [48]. Intensity should create a mild “pulling tension” but not pain. Flexibility training intensity and duration should be adjusted according to the level of flexibility, particularly for children with asthma recovering from an acute phase or exhibiting high airway reactivity.

In clinical settings, flexibility training is often integrated with aerobic and resistance exercises as part of pre- and post-exercise routines [103]. In pulmonary rehabilitation, flexibility training has demonstrated beneficial effects and has been shown to enhance exercise capacity and improve asthma symptom management [104, 105].

### Question 7: How should the exercise program be adjusted and progressed?

Recommendations: (1) Follow-up adjustment: the first follow-up should occur 1–2 weeks after implementation, with subsequent follow-ups every four weeks to optimize exercise content and intensity. (2) Progressive plan: in the first 4–6 weeks, gradually increase training duration by 5–10 minutes every 1–2 weeks. After one month of regular exercise, progressively adjust duration, frequency and/or intensity over the next 4–8 months. (3) Special periods: during respiratory infections or special periods such as flu season, pollen season or poor air quality, carefully evaluate the risks of exercise, increase follow-up frequency and adjust exercise type, intensity, frequency and duration under specialist guidance.

Evidence summary: To ensure the safety, effectiveness and sustainability of exercise interventions for children with asthma, the program should be adjusted based on clinical feedback and individual responses. Follow-up evaluations should be conducted within 1–2 weeks after starting exercise to assess subjective symptoms (e.g., shortness of breath and fatigue) and objective measures (e.g., peak expiratory flow and recovery time). Adjustments to intensity, frequency, or type of exercise should be made based on this feedback [106]. Existing research shows that early follow-up interventions significantly enhance adherence to individualized exercise programs, especially for children with chronic conditions such as asthma, by providing timely feedback to prevent exercise-induced symptoms [107]. Follow-up evaluations every four weeks are recommended to optimize exercise goals and strategies [106].

For progression, a “low starting point, gradual progression” approach is recommended for safety and effectiveness. The ACSM guidelines suggest gradually increasing training frequency, duration, or intensity to meet recommended fitness levels [48]. Research supports that progressive training helps improve aerobic capacity during rehabilitation, ultimately meeting health-related fitness standards [107]. Exercise programs must also consider environmental and pathological changes. During flu season, high pollen exposure, poor air quality, or following respiratory infections, the risks of exercise should be evaluated carefully and adjustments made. Increased follow-up frequency is recommended during these periods, with possible modifications such as lower-intensity indoor activities (e.g., walking, stretching, or breathing exercises) until intensity can be safely increased under healthcare provider guidance.

### Question 8: What preparations should be made before exercise?

Recommendations: (1) Lung function monitoring: use a handheld home spirometry device prior to exercise to obtain lung function data, enabling more effective monitoring of airflow limitation and facilitating personalized exercise recommendations by healthcare professionals. (2) Medication preparation: carry a SABA during exercise. For children with a history of EIB, preventive medication should be administered 10–20 minutes prior to exercise to minimize the risk of EIB and improve exercise tolerance. (3) Warm-up: warm-up for about 10 minutes with low-to-moderate intensity activities to help prevent EIB and improve exercise tolerance. (4) Exercise equipment: ensure appropriate equipment, including clothing, shoes and a water bottle. In cold environments, use a mask to warm and humidify inhaled air.

Evidence summary: Effective pre-exercise preparation is essential to ensure the safety of children with asthma and prevent EIB. A handheld home spirometry device is an effective tool for assessing lung function before exercise. This device provides more accurate data on airflow limitation, using reference values and z-scores to evaluate lung function. It also helps children better understand their respiratory status and enables healthcare professionals to track asthma control more precisely. By offering continuous monitoring, this device facilitates personalized exercise recommendations, ensuring safe participation in physical activity [108, 109].

Medication preparation is crucial, especially for children with a history of EIB. The Global Initiative for Asthma (GINA) recommends carrying a SABA for emergency use. For children sensitive to EIB, preventive medication taken 10–20 minutes before exercise significantly reduces EIB and improves exercise tolerance [19].

Warm-up activities are essential for optimizing cardiovascular function, improving airway reactivity and preventing exercise-related injuries. These activities are supported by substantial evidence [110, 111]. Warm-ups activate the neuromuscular system, raise core temperature and enhance bronchial smooth muscle adaptability, reducing the risk of EIB [112–114]. A warm-up of about 10 minutes is recommended which consist of three components. First, static stretching (e.g., chest expansion and leg stretches) to increase joint mobility and muscle elasticity, with each stretch held for < 20 seconds and a 10-second break to avoid reflexive muscle contraction. Second, dynamic warm-ups (e.g., slow jogging, lunges and jumping jacks) to activate the cardiovascular system and improve airway stability, with each set lasting around one minute, followed by a 10-second break and gradual increase in intensity. Third, passive warm-ups, where parents assist young or less compliant children with joint rotations and stretches to facilitate muscle activation and warm-up effects.

Finally, appropriate exercise equipment is crucial for safety and comfort. Children should wear well-fitting, breathable clothing and footwear and stay hydrated. In cold or dry environments, inhaling cold air can increase airway hyper-responsiveness. Wearing a humidifying mask is recommended to warm inhaled air, which has been shown to reduce EIB and minimize respiratory irritation [45, 112].

### Question 9: How should exercise intensity be monitored and evaluated?

Recommendations: (1) Intensity can be assessed using subjective methods like the RPE scale and the talk test, or by using relative measures such as heart rate reserve (HRR), VO_2_ reserve (VO_2_R), maximum heart rate (MHR), and maximum oxygen uptake (VO_2max_). Absolute measures, such as the metabolic equivalents (METs), can also be used to evaluate intensity. (2) Use wearable fitness trackers (e.g., fitness bands or smartwatches) to monitor real-time health data such as heart rate, respiratory rate, oxygen saturation, steps and activity level during exercise.

Evidence summary: Exercise intensity can be evaluated through two main approaches: subjective assessment and objective measurement. Subjective methods, such as the RPE and the talk test, rely on the perception of the individual of their effort during exercise and provides an estimate based on personal experience [115, 116]. In contrast, objective measurements are more precise and include the assessment of relative and absolute intensity. Relative intensity is typically calculated using methods like HRR, percentage of MHR, VO_2max_ and VO_2_R [48]. For instance, HRR is derived from the difference between maximal and resting heart rate, combined with a target intensity percentage. Absolute intensity is often evaluated using the MET, which quantifies energy expenditure during different activities [117]. Updated MET guidelines by Ainsworth et al. have provided standardized measures for common children’s activities, validated through multiple studies [118]. Recommended methods for calculating and evaluating exercise intensity [48], along with intensity grading [119–121], are presented in Tables 2 and 3.

**Table 2:** Exercise intensity calculation methods

Methods	Formula
HRR	Target HR = (HR_max_ − HR_rest_) × %Intensity + HR_rest_
VO_2_R	Target VO_2_ = (VO_2max_ − VO_2rest_) × %Intensity + VO_2rest_
HR_max_	Target HR = HR_max_ × %Intensity
VO_2max_	Target VO_2_ = VO_2max_ × %Intensity
METs	Target MET = (VO_2max_ ÷ 3.5) × %Intensity

*HR* heart rate, *VO*_*2*_ oxygen uptake, *HR*_*max/peak*_ maximum or peak heart rate, obtained through a maximal exercise stress test or estimated using predictive formulas, *HR*_*rest*_ resting heart rate, *HRR* heart rate reserve, the difference between HR_max_ and HR_rest_, *VO*_*2*_*R* oxygen uptake reserve, the difference between VO_2max_ and VO_2rest_, *VO*_*2max/peak*_ maximum or peak oxygen uptake, measured through a maximal graded exercise test or estimated via submaximal testing, *VO*_*2rest*_ resting oxygen uptake, *METs* metabolic equivalents

**Table 3:** Exercise intensity classification

Intensity	Subjective intensity	Relative intensity			Absolute intensity
	RPE	%HRR or %VO_2_R	%HR_max_	%VO_2__max_	METs
Very light	Very light (RPE < 9)	< 30	< 57	< 37	< 2
Light	Very light to fairly light (RPE 9–11)	30–39	57–63	37–45	2.0–2.9
Moderate	Fairly light to somewhat hard (RPE 12–13)	40–59	64–76	46–63	3.0–5.9
Vigorous	Somewhat hard to very hard (RPE 14–17)	60–89	77–95	64–90	6.0–8.7
Near-maximal to maximal	Very hard (RPE ≥ 18)	≥ 90	≥ 96	≥ 91	≥ 8.8

*RPE* rate of perceived exertion, *METs* metabolic equivalents, *HRR* heart rate reserve, *HR*_*max*_ maximum heart rate,  *VO*_*2max*_ maximum or peak oxygen uptake, *VO*_*2*_*R* oxygen uptake reserve

With the increasing use of wearable devices, real-time physiological data collection has become more accessible, allowing for personalized exercise adjustments. Wearable health trackers, such as fitness bands and smartwatches, can continuously monitor key indicators like heart rate, respiratory rate, physical activity, activity level, exercise duration and recovery status, offering comprehensive data to evaluate exercise intensity [122–124]. This detailed information enables more precise adjustments to exercise intensity based on current condition, ensuring that the exercise remains safe and effective. In addition, wearable devices can help ensure that children can participate in physical activities alongside their peers without experiencing undue discomfort. By monitoring exercise intensity in real-time, these devices help avoid excessive strain and promote a positive exercise experience, fostering social interaction and inclusivity while maintaining health and safety.

### Question 10: What aspects should exercise outcomes be evaluated from?

Recommendations: (1) Physical fitness: assess nutritional and physical development of the child by measuring height, weight and calculating body mass index (BMI)-Z. (2) Asthma control: regularly use spirometry to assess the impact of exercise on asthma control. When utilizing asthma control questionnaires, longitudinal tracking of scores is recommended to provide more reliable insights into symptom perception over time. (3) Cardiopulmonary function: assess cardiopulmonary function through maximal or submaximal exercise testing to monitor improvements in endurance and fitness. (4) Quality of life and psychological health: assess improvements in psychological state and well-being of the child using asthma-specific quality of life questionnaires and anxiety/depression scales.

Evidence summary: A systematic exercise intervention should be paired with a multidimensional evaluation system to assess its effects on physical health, respiratory function, cardiopulmonary fitness and psychological well-being in children with asthma. Recent research emphasizes combining objective measures with subjective experiences for individualized feedback and intervention adjustments [125].

For physical fitness, the BMI-Z score, which adjusts for age and gender, is a more standardized indicator than BMI for evaluating body composition and nutritional status. Regular exercise not only enhances physical activity but also helps regulate body weight and prevents obesity-related airway inflammation [126].

Assessment of lung function and asthma control is critical for determining whether exercise interventions effectively reduce airway hyperresponsiveness [127]. Pulmonary function tests (e.g., FEV_1_ and PEF) are widely used objective tools, with their variability reflecting changes in airway patency and responsiveness. In addition, handheld spirometry devices can offer more accurate assessments of lung function prior to exercise by providing reference-based data and z-scores. This helps healthcare professionals track asthma control with greater precision and tailor exercise recommendations. In parallel, asthma control questionnaires (e.g., C-ACT and asthma control test) provide standardized subjective scores based on symptom frequency, nighttime awakenings and rescue medication use. Longitudinal tracking of questionnaire scores may provide more meaningful insights into symptom perception, as single assessments often yield limited objective information [128, 129].

Enhancing cardiopulmonary endurance is a key goal of exercise interventions. VO_2max_ is considered the gold standard for evaluating cardiopulmonary function, reflecting the ability of the body to utilize oxygen. However, VO_2max_ testing requires specialized equipment (e.g., gas analyzers and treadmills), rendering the ability of children to complete due to baseline conditions or adherence issues. In these cases, submaximal tests Like the 6MWT are reliable alternatives, especially in resource-limited settings or for children with lower physical capacity [48, 130].

In addition to physiological measures, psychological state and quality of life are vital for evaluating exercise outcomes. Studies emphasize the emotional and social challenges faced by children with asthma. Tools such as the pediatric asthma quality of life questionnaire (PAQLQ), child anxiety scale and children’s depression self-rating scale are commonly used to assess changes in psychological well-being and quality of life [28, 39, 44].

### Question 11: How should exercise-induced bronchoconstriction be identified?

Recommendations: EIB should be considered based on characteristic symptoms, such as cough, wheezing, dyspnea and chest tightness, occurring within 15 minutes after 5–8 minutes of intense exercise. Diagnosis should be confirmed with exercise challenge testing and spirometry, demonstrating a ≥ 10% decrease in FEV_1_. Spirometry should be performed as soon as possible after exercise, particularly in younger children.

Evidence summary: The prevalence of EIB in the general population ranges from 5% to 20%, but is significantly higher among children and adolescents with asthma, reaching up to 46% [131, 132]. EIB typically occurs following intense exercise, characterized by airway narrowing that leads to symptoms such as shortness of breath, wheezing, chest tightness and coughing. EIB usually develops within 15 minutes after 5–8 minutes of intensive aerobic training and typically resolves within 60 minutes [133]. However, because the clinical symptoms of EIB overlap with those of other respiratory conditions, including asthma and upper respiratory infections, a definitive diagnosis based solely on symptoms is often challenging. Therefore, objective diagnostic methods are essential for accurate identification.

The ATS recommends exercise challenge testing as the standard diagnostic method for EIB. This test is typically conducted in a controlled environment where dry, cold air is used to simulate the conditions that commonly trigger EIB during exercise. A decrease in FEV_1_ of ≥ 10% post-exercise is considered diagnostic for EIB. Spirometry should be performed soon after exercise, especially in younger children (ages < 8), as EIB may have a quicker onset and could resolve within five minutes after cessation of exercise. In cases of breakthrough EIB, FEV_1_ decreases can occur during exercise itself. EIB severity is classified according to the degree of FEV_1_ reduction: mild EIB is defined as a 10% to < 25% decrease in FEV_1_, moderate EIB as a 25% to < 50% reduction and severe EIB as a ≥ 50% drop in FEV_1_ [11, 133]. In addition, hyperventilation and dry saline inhalation tests can also be used to diagnose EIB by simulating the airway dehydration that occurs during exercise [134]. Notably, FeNO testing has emerged as a promising tool for diagnosing EIB. By measuring airway inflammation, FeNO helps distinguish EIB from other conditions, particularly in pediatric patients, providing more direct indicators of airway inflammation to assist in clinical diagnosis [135–137].

### Question 12: How should exercise-induced bronchoconstriction be prevented and managed?

Recommendations: (1) Pharmacological prevention and treatment: fast-acting bronchodilators such as SABAs are first-line for EIB and should be used 5–20 minutes before exercise. If symptoms persist or SABA is required daily or more frequently, daily ICS or leukotriene receptor antagonists (LTRAs) are recommended for long-term management. Long-acting beta2-agonist (LABA) monotherapy is not recommended and should be used in combination with ICS when additional control is necessary. (2) Non-pharmacological interventions: warm-up exercises should be performed before physical activity and patients should avoid exercise in cold, dry, or polluted environments. Masks that warm and humidify the air may help reduce the occurrence of EIB. (3) Lifestyle modifications: regular exercise, weight control and maintaining overall physical fitness are recommended to reduce the occurrence of EIB.

Evidence summary: Pharmacological treatment and adherence to therapy are the cornerstone of asthma control and are paramount in preventing EIB. Fast-acting bronchodilators such as SABAs are first-line treatment for EIB, as they rapidly alleviate bronchoconstriction by stimulating β_2_-receptors on airway smooth muscle, causing bronchodilation; they may also reduce mast cell degranulation [138]. For prevention, SABAs are typically inhaled 5–20 minutes before exercise, with around 15 minutes being optimal. However, frequent or daily use of SABAs can lead to β_2_-receptor desensitization and reduced duration of protection. Therefore, SABAs are generally recommended for intermittent use. Patients requiring daily or more frequent SABA use should initiate controller therapy. Daily ICS are considered the most effective anti-inflammatory agents for managing persistent symptoms and improving airway inflammation associated with EIB [11]. LTRAs represent an alternative or adjunctive option for long-term management, blocking leukotriene-mediated inflammation, reducing inflammatory mediator release and providing sustained broncho-protection without causing tolerance [139]. For patients with more severe or difficult-to-control EIB, combination therapy with ICS and LTRA or ICS and LABAs may be considered [11, 140]. The risks associated with daily LABA monotherapy, including increased asthma-related mortality and higher exacerbation rates, outweigh its benefits, such as reduced dyspnea and decreased need for SABAs. Given the availability of safer and more effective treatment options, LABA monotherapy is not recommended [11]. Regular monitoring is necessary to assess treatment effectiveness and detect potential adverse effects [139].

The GINA recommends adequate warm-up exercises before physical activity as an effective non-pharmacological treatment to prevent EIB [12]. Gradually increasing exercise intensity allows the respiratory system to adapt to increasing ventilatory demands, reducing the risk of bronchoconstriction [141]. Furthermore, patients with EIB should avoid vigorous exercise in cold, dry, or polluted environments, as these factors exacerbate EIB symptoms. Research suggests that wearing masks that warm and humidify the air may reduce the occurrence of EIB [45]. While high-quality evidence is limited, some small-scale studies have demonstrated the potential benefits of this approach.

Improving lifestyle habits is an important component of EIB management. Increasing physical fitness and endurance has been shown to significantly reduce the occurrence of EIB. Regular exercise can reduce airway inflammation, promote symptom relief and contribute to better asthma control [142, 143]. Maintaining a healthy weight and preventing obesity are also effective strategies to mitigate EIB symptoms [138]. Through exercise training, patients can improve their physical capacity and reduce both the frequency and severity of EIB episodes [142]. While low-sodium diets and supplementation with fish oil and vitamin C may be beneficial for some patients, there is insufficient evidence to make definitive recommendations [11, 144]. However, studies have shown that breathing control techniques (e.g., yoga and supervised breathing training) can not only alleviate EIB symptoms but also reduce drug dependence, lower anxiety and depressive symptoms associated with EIB and significantly improve quality of life [140].

## Conclusions

In conclusion, physical exercise is a valuable component of asthma management for children, providing significant health benefits when asthma is well-controlled. With proper pharmacological treatment and individualized exercise plans, most children can safely participate in physical activity, improving lung function, cardiovascular fitness and overall well-being. Exercise also supports mental health and social development. The key to safe participation is maintaining asthma control through symptom monitoring and adjusting exercise intensity based on asthma status and environmental factors. Overall, physical activity should be encouraged as an integral part of asthma care to enhance both physical and psychological outcomes in children with asthma.

## Data Availability

Not applicable.

## References

[1] Zheng J, Jin YJ, Wang CH, Feng C, Lai XY, Hua SQ, et al. Global, regional, and national epidemiology of allergic diseases in children from 1990 to 2021: findings from the global burden of disease study 2021. BMC Pulm Med. 2025;25:54. 39891163 10.1186/s12890-025-03518-y PMC11786411

[2] Yuan L, Tao J, Wang J, She W, Zou Y, Li R, et al. Global, regional, national burden of asthma from 1990 to 2021, with projections of incidence to 2050: a systematic analysis of the global burden of disease study 2021. EClinicalMedicine. 2025;80:103051. 39867965 10.1016/j.eclinm.2024.103051 PMC11764843

[3] Pijnenburg MW, Frey U, De Jongste JC, Saglani S. Childhood asthma: pathogenesis and phenotypes. Eur Respir J. 2022;59:2100731. 34711541 10.1183/13993003.00731-2021

[4] Schoettler N, Strek ME. Recent advances in severe asthma: from phenotypes to personalized medicine. Chest. 2020;157:516–28. 31678077 10.1016/j.chest.2019.10.009 PMC7609962

[5] Aggarwal B, Mulgirigama A, Berend N. Exercise-induced bronchoconstriction: prevalence, pathophysiology, patient impact, diagnosis and management. NPJ Prim Care Respir Med. 2018;28:31. 30108224 10.1038/s41533-018-0098-2 PMC6092370

[6] Lu KD, Manoukian K, Radom-Aizik S, Cooper DM, Galant SP. Obesity, asthma, and exercise in child and adolescent health. Pediatr Exerc Sci. 2016;28:264–74. 26618409 10.1123/pes.2015-0122 PMC5904022

[7] Eichenberger PA, Diener SN, Kofmehl R, Spengler CM. Effects of exercise training on airway hyperreactivity in asthma: a systematic review and meta-analysis. Sports Med. 2013;43:1157–70. 23846823 10.1007/s40279-013-0077-2

[8] Lin N, Huang Z, Li J, Dong J, Yan X, Chen Z, et al. Effects of high-intensity interval training on exercise capacity and asthma-related outcomes in children: a systematic review. Allergy. 2025;80:1464–7. 39347650 10.1111/all.16336

[9] Zhang YF, Yang LD. Exercise training as an adjunctive therapy to montelukast in children with mild asthma: a randomized controlled trial. Medicine. 2019;98:e14046. 30633202 10.1097/MD.0000000000014046 PMC6336542

[10] Ma Q, Lu M, Yang Q, Gong F, Zhou L, Xu D. Effects of aerobic exercise-based pulmonary rehabilitation on quality of life in pediatric asthma: a systematic review and meta-analysis. Heart Lung. 2025;69:11–30. 39276534 10.1016/j.hrtlng.2024.09.005

[11] Parsons JP, Hallstrand TS, Mastronarde JG, Kaminsky DA, Rundell KW, Hull JH, et al. An official American Thoracic Society clinical practice guideline: exercise-induced bronchoconstriction. Am J Respir Crit Care Med. 2013;187:1016–27. 23634861 10.1164/rccm.201303-0437ST

[12] Reddel HK, Bacharier LB, Bateman ED, Brightling CE, Brusselle GG, Buhl R, et al. Global initiative for asthma strategy 2021: executive summary and rationale for key changes. Eur Respir J. 2022;59:2102730. 34667060 10.1183/13993003.02730-2021 PMC8719459

[13] Durieux N, Vandenput S, Pasleau F. OCEBM levels of evidence system. Rev Med Liege. 2013;68:644–9 (**in French**). 24564030

[14] Weisgerber M, Webber K, Meurer J, Danduran M, Berger S, Flores G. Moderate and vigorous exercise programs in children with asthma: safety, parental satisfaction, and asthma outcomes. Pediatr Pulmonol. 2008;43:1175–82. 19003892 10.1002/ppul.20895

[15] Price OJ, Simpson AJ. Exercise and asthma–trigger or treatment? Respir Med. 2023;213:107247. 37086818 10.1016/j.rmed.2023.107247

[16] Freeman AT, Staples KJ, Wilkinson TMA. Defining a role for exercise training in the management of asthma. Eur Respir Rev. 2020;29:190106. 32620584 10.1183/16000617.0106-2019 PMC9489044

[17] Lu KD, Forno E. Exercise and lifestyle changes in pediatric asthma. Curr Opin Pulm Med. 2020;26:103–11. 31652153 10.1097/MCP.0000000000000636 PMC7094764

[18] Wanrooij VH, Willeboordse M, Dompeling E, van de Kant KD. Exercise training in children with asthma: a systematic review. Br J Sports Med. 2014;48:1024–31. 23525551 10.1136/bjsports-2012-091347

[19] Venkatesan P. 2025 GINA report for asthma. Lancet Respir Med. 2025;13:E41–2. 10.1016/S2213-2600(25)00242-5 40582369

[20] Bonini M, Palange P. Exercise-induced bronchoconstriction: new evidence in pathogenesis, diagnosis and treatment. Asthma Res Pract. 2015;1:2. 27965757 10.1186/s40733-015-0004-4 PMC4970375

[21] Nyenhuis SM, Kahwash B, Cooke A, Gregory KL, Greiwe J, Nanda A. Recommendations for physical activity in asthma: a work group report of the AAAAI sports, exercise, and fitness committee. J Allergy Clin Immunol Pract. 2022;10:433–43. 34844909 10.1016/j.jaip.2021.10.056

[22] Papi A, Ferreira DS, Agache I, Baraldi E, Beasley R, Brusselle G, et al. European respiratory society short guidelines for the use of as-needed ICS/formoterol in mild asthma. Eur Respir J. 2023;62:2300047. 37678955 10.1183/13993003.00047-2023

[23] Hengeveld VS, Keijzer PB, Diamant Z, Thio BJ. An algorithm for strategic continuation or restriction of asthma medication prior to exercise challenge testing in childhood exercise induced bronchoconstriction. Front Pediatr. 2022;10:800193. 35273926 10.3389/fped.2022.800193 PMC8902070

[24] Anderson SD, Kippelen P. Assessment and prevention of exercise-induced bronchoconstriction. Br J Sports Med. 2012;46:391–6. 22247297 10.1136/bjsports-2011-090810

[25] Weiler JM, Brannan JD, Randolph CC, Hallstrand TS, Parsons J, Silvers W, et al. Exercise-induced bronchoconstriction update-2016. J Allergy Clin Immunol. 2016;138:1292–5.e36. 27665489 10.1016/j.jaci.2016.05.029

[26] Abdelbasset WK, Alsubaie SF, Tantawy SA, Abo Elyazed TI, Kamel DM. Evaluating pulmonary function, aerobic capacity, and pediatric quality of life following a 10-week aerobic exercise training in school-aged asthmatics: a randomized controlled trial. Patient Prefer Adherence. 2018;12:1015–23. 29942118 10.2147/PPA.S159622 PMC6007206

[27] Liu Y, Zhao Y, Liu F, Liu L. Effects of physical exercises on pulmonary rehabilitation, exercise capacity, and quality of life in children with asthma: a meta-analysis. Evid Based Complement Alternat Med. 2021;2021:5104102. 34976094 10.1155/2021/5104102 PMC8718301

[28] Liu F, Liu YR, Liu L. Effect of exercise rehabilitation on exercise capacity and quality of life in children with bronchial asthma: a systematic review. Chin J Contemp Pediatr. 2021;23:1050–7. 10.7499/j.issn.1008-8830.2104124 PMC8549640 34719422

[29] Welsh L, Kemp JG, Roberts RG. Effects of physical conditioning on children and adolescents with asthma. Sports Med. 2005;35:127–41. 15707377 10.2165/00007256-200535020-00003

[30] Andrade LB, Britto MC, Lucena-Silva N, Gomes RG, Figueroa JN. The efficacy of aerobic training in improving the inflammatory component of asthmatic children. Randomized trial. Respir Med. 2014;108:1438–45. 25231109 10.1016/j.rmed.2014.07.009

[31] Francisco CO, Bhatawadekar SA, Babineau J, Reid WD, Yadollahi A. Effects of physical exercise training on nocturnal symptoms in asthma: systematic review. PLoS ONE. 2018;13:e0204953. 30346958 10.1371/journal.pone.0204953 PMC6197640

[32] Zhou L, Xu H. Feasibility of exercise therapy for children with asthma: a meta-analysis. Front Cell Dev Biol. 2023;11:1192929. 37492220 10.3389/fcell.2023.1192929 PMC10364120

[33] Gomes EL, Carvalho CR, Peixoto-Souza FS, Teixeira-Carvalho EF, Mendonça JF, Stirbulov R, et al. Active video game exercise training improves the clinical control of asthma in children: randomized controlled trial. PLoS ONE. 2015;10:e0135433. 26301706 10.1371/journal.pone.0135433 PMC4547724

[34] Latorre-Román PÁ, Navarro-Martínez AV, García-Pinillos F. The effectiveness of an indoor intermittent training program for improving lung function, physical capacity, body composition and quality of life in children with asthma. J Asthma. 2014;51:544–51. 24471516 10.3109/02770903.2014.888573

[35] Sanz-Santiago V, Diez-Vega I, Santana-Sosa E, Lopez Nuevo C, Iturriaga Ramirez T, Vendrusculo FM, et al. Effect of a combined exercise program on physical fitness, lung function, and quality of life in patients with controlled asthma and exercise symptoms: a randomized controlled trial. Pediatr Pulmonol. 2020;55:1608–16. 32353218 10.1002/ppul.24798

[36] van Veldhoven NH, Vermeer A, Bogaard JM, Hessels MG, Wijnroks L, Colland VT, et al. Children with asthma and physical exercise: effects of an exercise programme. Clin Rehabil. 2001;15:360–70. 11518437 10.1191/026921501678310162

[37] Carew C, Cox DW. Laps or lengths? The effects of different exercise programs on asthma control in children. J Asthma. 2018;55:877–81. 28872938 10.1080/02770903.2017.1373806

[38] Ramachandran HJ, Jiang Y, Shan CH, Tam WWS, Wang W. A systematic review and meta-analysis on the effectiveness of swimming on lung function and asthma control in children with asthma. Int J Nurs Stud. 2021;120:103953. 34051586 10.1016/j.ijnurstu.2021.103953

[39] Crosbie A. The effect of physical training in children with asthma on pulmonary function, aerobic capacity and health-related quality of life: a systematic review of randomized control trials. Pediatr Exerc Sci. 2012;24:472–89. 22971562 10.1123/pes.24.3.472

[40] Fanelli A, Cabral AL, Neder JA, Martins MA, Carvalho CR. Exercise training on disease control and quality of life in asthmatic children. Med Sci Sports Exerc. 2007;39:1474–80. 17805077 10.1249/mss.0b013e3180d099ad

[41] Westergren T, Aagaard H, Hall EOC, Ludvigsen MS, Fegran L, Robstad N, et al. Physical activity enforces well-being or shame in children and adolescents with asthma: a meta-ethnography. Inquiry. 2024;61:469580241290086. 39497650 10.1177/00469580241290086 PMC11536505

[42] Voorend-van Bergen S, Vaessen-Verberne AA, de Jongste JC, Pijnenburg MW. Asthma control questionnaires in the management of asthma in children: a review. Pediatr Pulmonol. 2015;50:202–8. 25187271 10.1002/ppul.23098

[43] Feng Y, Shang YX. Role of peak expiratory flow in the assessment and management of asthma in children. Chin J Contemp Pediatr. 2021;23:645–9. 10.7499/j.issn.1008-8830.2101134 PMC8213995 34130789

[44] Jiang J, Zhang D, Huang Y, Wu Z, Zhang W. Exercise rehabilitation in pediatric asthma: a systematic review and network meta-analysis. Pediatr Pulmonol. 2022;57:2915–27. 36103241 10.1002/ppul.26134

[45] Morton AR, Fitch KD. Australian association for exercise and sports science position statement on exercise and asthma. J Sci Med Sport. 2011;14:312–6. 21440499 10.1016/j.jsams.2011.02.009

[46] Manti S, Licari A, Leonardi S, Marseglia GL. Management of asthma exacerbations in the paediatric population: a systematic review. Eur Respir Rev. 2021;30:200367. 34261742 10.1183/16000617.0367-2020 PMC9488496

[47] Reddel HK, FitzGerald JM, Bateman ED, Bacharier LB, Becker A, Brusselle G, et al. GINA 2019: a fundamental change in asthma management: treatment of asthma with short-acting bronchodilators alone is no longer recommended for adults and adolescents. Eur Respir J. 2019;53:1901046. 31249014 10.1183/13993003.01046-2019

[48] American College of Sports Medicine, Liguori G, Feito Y, Fountaine C, Roy B. ACSM's guidelines for exercise testing and prescription. Philadelphia: Wolters Kluwer; 2022.

[49] Guazzi M, Adams V, Conraads V, Halle M, Mezzani A, Vanhees L, et al. EACPR/AHA Scientific Statement. Clinical recommendations for cardiopulmonary exercise testing data assessment in specific patient populations. Circulation. 2012;126:2261–74. 10.1161/CIR.0b013e31826fb946 PMC4777325 22952317

[50] American Thoracic Society, American College of Chest Physicians. ATS/ACCP Statement on cardiopulmonary exercise testing. Am J Respir Crit Care Med. 2003;167:211–77. 12524257 10.1164/rccm.167.2.211

[51] Zampogna E, Ambrosino N, Centis R, Cherubino F, Migliori GB, Pignatti P, et al. Minimal clinically important difference of the 6-min walking test in patients with asthma. Int J Tuberc Lung Dis. 2021;25:215–21. 33688810 10.5588/ijtld.20.0928

[52] Liu Y, Du Q, Jiang Y. The effect of virtual reality technology in exercise and lung function of patients with chronic obstructive pulmonary disease: a systematic review and meta-analysis. Worldviews Evid Based Nurs. 2024;21:307–17. 38297408 10.1111/wvn.12698

[53] ATS Committee on Proficiency Standards for Clinical Pulmonary Function Laboratories. ATS statement: guidelines for the six-minute walk test. Am J Respir Crit Care Med. 2002;166:111–7. 10.1164/ajrccm.166.1.at1102 12091180

[54] Tharumakunarajah R, Lee A, Hawcutt DB, Harman NL, Sinha IP. The impact of malnutrition on the developing lung and long-term lung health: a narrative review of global literature. Pulm Ther. 2024;10:155–70. 38758409 10.1007/s41030-024-00257-z PMC11282003

[55] Freedman DS, Goodwin Davies AJ, Phan TT, Cole FS, Pajor N, Rao S, et al. Measuring BMI change among children and adolescents. Pediatr Obes. 2022;17:e12889. 35064761 10.1111/ijpo.12889 PMC11135243

[56] Cavaggioni L, Gilardini L, Croci M, Formenti D, Merati G, Bertoli S. The usefulness of integrative neuromuscular training to counteract obesity: a narrative review. Int J Obes. 2024;48:22–32. 10.1038/s41366-023-01392-4 PMC10746545 37775520

[57] Vandoni M, Marin L, Cavallo C, Gatti A, Grazi R, Albanese I, et al. Poor motor competence affects functional capacities and healthcare in children and adolescents with obesity. Sports. 2024;12:44. 38393264 10.3390/sports12020044 PMC10891969

[58] Granacher U, Muehlbauer T, Maestrini L, Zahner L, Gollhofer A. Can balance training promote balance and strength in prepubertal children? J Strength Cond Res. 2011;25:1759–66. 21386732 10.1519/JSC.0b013e3181da7886

[59] Clarke R, Heath G, Nagakumar P, Pattison H, Farrow C. “He’s not fat, he just has asthma”: a qualitative study exploring weight management in families living with pediatric asthma. J Asthma. 2022;59:1750–7. 34470559 10.1080/02770903.2021.1975739

[60] Koskela-Staples NC, Yourell JL, Fedele DA, Doty J. Physical activity engagement: perspectives from adolescents with comorbid asthma and overweight/obesity and their caregivers. J Pediatr Psychol. 2023;48:707–19. 37316999 10.1093/jpepsy/jsad035 PMC10467644

[61] Rafiei Milajerdi H, Sheikh M, Najafabadi MG, Saghaei B, Naghdi N, Dewey D. The effects of physical activity and exergaming on motor skills and executive functions in children with autism spectrum disorder. Games Health J. 2021;10:33–42. 33370161 10.1089/g4h.2019.0180

[62] Sharp CA, McNarry MA, Eddolls WTB, Koorts H, Winn CON, MacKintosh KA. Identifying facilitators and barriers for adolescents participating in a school-based HIIT intervention: the exercise for asthma with commando Joe’s® (X4ACJ) programme. BMC Public Health. 2020;20:609. 32357869 10.1186/s12889-020-08740-3 PMC7195754

[63] Bolger LE, Bolger LA, O’Neill C, Coughlan E, O’Brien W, Lacey S, et al. Global levels of fundamental motor skills in children: a systematic review. J Sports Sci. 2021;39:717–53. 33377417 10.1080/02640414.2020.1841405

[64] Hu D, Zhou S, Crowley-McHattan ZJ, Liu Z. A comparative study of the physical activity guidelines for children and adolescents from five countries and WHO. Front Public Health. 2024;12:1421843. 39071153 10.3389/fpubh.2024.1421843 PMC11272551

[65] Parrish AM, Tremblay MS, Carson S, Veldman SLC, Cliff D, Vella S, et al. Comparing and assessing physical activity guidelines for children and adolescents: a systematic literature review and analysis. Int J Behav Nutr Phys Act. 2020;17:16. 32041635 10.1186/s12966-020-0914-2 PMC7011603

[66] Chaput JP, Willumsen J, Bull F, Chou R, Ekelund U, Firth J, et al. 2020 WHO guidelines on physical activity and sedentary behaviour for children and adolescents aged 5–17 years: summary of the evidence. Int J Behav Nutr Phys Act. 2020;17:141. 33239009 10.1186/s12966-020-01037-z PMC7691077

[67] Farr JN, Laddu DR, Going SB. Exercise, hormones and skeletal adaptations during childhood and adolescence. Pediatr Exerc Sci. 2014;26:384–91. 25372373 10.1123/pes.2014-0077 PMC4356169

[68] Carlsen KH, Engh G, Mørk M. Exercise-induced bronchoconstriction depends on exercise load. Respir Med. 2000;94:750–5. 10955749 10.1053/rmed.2000.0809

[69] Ora J, de Marco P, Gabriele M, Cazzola M, Rogliani P. Exercise-induced asthma: managing respiratory issues in athletes. J Funct Morphol Kinesiol. 2024;9:15. 38249092 10.3390/jfmk9010015 PMC10801521

[70] Tikkakoski AP, Tikkakoski A, Sipilä K, Kivistö JE, Huhtala H, Kähönen M, et al. Exercise-induced bronchoconstriction is associated with air humidity and particulate matter concentration in preschool children. Pediatr Pulmonol. 2023;58:996–1003. 36530015 10.1002/ppul.26284

[71] Tikkakoski AP, Reini M, Sipilä K, Kivistö JE, Karjalainen J, Kähönen M, et al. Association of temperature and absolute humidity with incidence of exercise-induced bronchoconstriction in children. Acta Paediatr. 2024;113:1942–8. 38780114 10.1111/apa.17295

[72] Tikkakoski AP, Tikkakoski A, Kivistö JE, Huhtala H, Sipilä K, Karjalainen J, et al. Association of air humidity with incidence of exercise-induced bronchoconstriction in children. Pediatr Pulmonol. 2019;54:1830–6. 31393065 10.1002/ppul.24471

[73] Stensrud T, Berntsen S, Carlsen KH. Humidity influences exercise capacity in subjects with exercise-induced bronchoconstriction (EIB). Respir Med. 2006;100:1633–41. 16446080 10.1016/j.rmed.2005.12.001

[74] Pan R, Wang X, Yi W, Wei Q, Gao J, Xu Z, et al. Interactions between climate factors and air quality index for improved childhood asthma self-management. Sci Total Environ. 2020;723:137804. 32213400 10.1016/j.scitotenv.2020.137804

[75] United Nations Children’s Fund (UNICEF). Clear the Air for Children. 2016. https://www.unicef.org/publications/index92957.html. Accessed 28 May 2025.

[76] U.S. Environmental Protection Agency. Air quality index - a guide to air quality and your health, 2014. EPA-456/F-14-002. 2014. http://www.airnow.gov/sites/default/files/2018-04/aqi_brochure_02_14_0.pdf. Accessed 28 May 2025.

[77] Tsai YG, Chio CP, Yang KD, Lin CH, Yeh YP, Chang YJ, et al. Long-term PM_2.5_ exposure is associated with asthma prevalence and exhaled nitric oxide levels in children. Pediatr Res. 2025;97:370–7. 38263452 10.1038/s41390-023-02977-5

[78] Hu YB, Chen YT, Liu SJ, Jiang F, Wu MQ, Yan CH, et al. Increasing prevalence and influencing factors of childhood asthma: a cross-sectional study in Shanghai, China. World J Pediatr. 2021;17:419–28. 34110593 10.1007/s12519-021-00436-x

[79] Grande AJ, Silva V, Andriolo BN, Riera R, Parra SA, Peccin MS. Water-based exercise for adults with asthma. Cochrane Database Syst Rev. 2014;2014:CD010456. 25032820 10.1002/14651858.CD010456.pub2 PMC11252722

[80] Sahakian NM, Park JH, Cox-Ganser JM. Dampness and mold in the indoor environment: implications for asthma. Immunol Allergy Clin North Am. 2008;28:485–505,vii. 18572103 10.1016/j.iac.2008.03.009

[81] Boyle RJ, Tang ML. Environment and asthma. N Engl J Med. 2004;351:2654–5. 15602028 10.1056/NEJM200412163512518

[82] Rodrigues D, Padez C, Machado-Rodrigues AM. Parental perception of barriers to children’s participation in sports: biological, social, and geographic correlates of Portuguese children. J Phys Act Health. 2019;16:595–600. 31195909 10.1123/jpah.2018-0390

[83] Wang H, Swain S, Luo J, Blake H, Chattopadhyay K. Barriers and facilitators to physical activity among ethnic Chinese children: a qualitative systematic review. JBI Evid Synth. 2020;18:2445–511. 32833787 10.11124/JBISRIR-D-19-00154

[84] Xu H, Wen LM, Rissel C. Associations of parental influences with physical activity and screen time among young children: a systematic review. J Obes. 2015;2015:546925. 25874123 10.1155/2015/546925 PMC4383435

[85] Jones T, Baque E, O’Grady KA, Kohler BE, Goyal V, McCallum GB, et al. Experiences of children with bronchiectasis and their parents in a novel play-based therapeutic exercise programme: a qualitative analysis. BMJ Open. 2024;14:e078994. 39089712 10.1136/bmjopen-2023-078994 PMC11293381

[86] Petersen TL, Møller LB, Brønd JC, Jepsen R, Grøntved A. Association between parent and child physical activity: a systematic review. Int J Behav Nutr Phys Act. 2020;17:67. 32423407 10.1186/s12966-020-00966-z PMC7236180

[87] Watson A, Timperio A, Brown H, Best K, Hesketh KD. Effect of classroom-based physical activity interventions on academic and physical activity outcomes: a systematic review and meta-analysis. Int J Behav Nutr Phys Act. 2017;14:114. 28841890 10.1186/s12966-017-0569-9 PMC5574081

[88] Kliziene I, Cizauskas G, Sipaviciene S, Aleksandraviciene R, Zaicenkoviene K. Effects of a physical education program on physical activity and emotional well-being among primary school children. Int J Environ Res Public Health. 2021;18:7536. 34299987 10.3390/ijerph18147536 PMC8304760

[89] Galvez MP, McCarthy K, Sarabu C, Mears A. The built environment and childhood obesity. Pediatr Clin North Am. 2024;71:831–43. 39343496 10.1016/j.pcl.2024.06.004 PMC11443065

[90] Ige TJ, DeLeon P, Nabors L. Motivational interviewing in an obesity prevention program for children. Health Promot Pract. 2017;18:263–74. 27199150 10.1177/1524839916647330

[91] Suire KB, Kavookjian J, Wadsworth DD. Motivational interviewing for overweight children: a systematic review. Pediatrics. 2020;146:e20200193. 33055225 10.1542/peds.2020-0193

[92] Teixeira PJ, Carraça EV, Markland D, Silva MN, Ryan RM. Exercise, physical activity, and self-determination theory: a systematic review. Int J Behav Nutr Phys Act. 2012;9:78. 22726453 10.1186/1479-5868-9-78 PMC3441783

[93] Cowie E, White K, Hamilton K. Physical activity and parents of very young children: the role of beliefs and social-cognitive factors. Br J Health Psychol. 2018;23:782–803. 29761636 10.1111/bjhp.12316

[94] Martin JJ, McCaughtry N, Flory S, Murphy A, Wisdom K. Using social cognitive theory to predict physical activity and fitness in underserved middle school children. Res Q Exerc Sport. 2011;82:247–55. 21699104 10.1080/02701367.2011.10599752

[95] Sheng J, Shi P, Sun J, Feng X. Predictors of physical activity behavior transitions in children and adolescents: a systematic review based on a transtheoretical model. J Healthc Eng. 2023;2023:5786841. 36824408 10.1155/2023/5786841 PMC9943610

[96] Neder JA, Nery LE, Silva AC, Cabral AL, Fernandes AL. Short-term effects of aerobic training in the clinical management of moderate to severe asthma in children. Thorax. 1999;54:202–6. 10325894 10.1136/thx.54.3.202 PMC1745434

[97] Bull FC, Al-Ansari SS, Biddle S, Borodulin K, Buman MP, Cardon G, et al. World Health Organization 2020 guidelines on physical activity and sedentary behaviour. Br J Sports Med. 2020;54:1451–62. 33239350 10.1136/bjsports-2020-102955 PMC7719906

[98] Khodashenas E, Bakhtiari E, Sohrabi M, Mozayani A, Arabi M, Haghighi VV, et al. The effect of a selective exercise program on motor competence and pulmonary function of asthmatic children: a randomized clinical trial. Int J Pediatr. 2019;7:9711–7.

[99] Eddolls WTB, McNarry MA, Stratton G, Winn CON, MacKintosh KA. High-intensity interval training interventions in children and adolescents: a systematic review. Sports Med. 2017;47:2363–74. 28643209 10.1007/s40279-017-0753-8 PMC5633633

[100] Visser E, Jong KD, van Zutphen T, Kerstjens HAM, Ten Brinke A. Muscle function in moderate to severe asthma: association with clinical outcomes and inflammatory markers. J Allergy Clin Immunol Pract. 2023;11:1439–47.e3. 36693537 10.1016/j.jaip.2022.12.043

[101] World Health Organization. WHO guidelines on physical activity and sedentary behaviour. Geneva: World Health Organization; 2020. 33369898

[102] Olenich S, Waterworth G, Badger GJ, Levy B, Israel E, Langevin HM. Flexibility and strength training in asthma: a pilot study. J Asthma. 2018;55:1376–83. 29420095 10.1080/02770903.2017.1414236

[103] Simão R, Lemos A, Salles B, Leite T, Oliveira É, Rhea M, et al. The influence of strength, flexibility, and simultaneous training on flexibility and strength gains. J Strength Cond Res. 2011;25:1333–8. 21386731 10.1519/JSC.0b013e3181da85bf

[104] Chushkin MI, Popova LA, Mandrykin SY, Kaprina NL. Use of exercise tests and physical training in pulmonary rehabilitation. Vopr Kurortol Fizioter Lech Fiz Kult. 2021;98:64–70. 33605132 10.17116/kurort20219801164

[105] Li W, Liu T, Yao M, Yu R, Shu M, Zhang M, et al. Effect of interesting respiratory rehabilitation training for the treatment of refractory *Mycoplasma pneumoniae* pneumonia in children. BMC Infect Dis. 2023;23:561. 37641025 10.1186/s12879-023-08513-4 PMC10464032

[106] Shen K, Zhu Z. Expert consensus on exercise prescription for asthmatic children in China. Chin J Appl Clin Pediatr. 2022;37:563–71.

[107] Westergren T, Fegran L, Nilsen T, Haraldstad K, Kittang OB, Berntsen S. Active play exercise intervention in children with asthma: a pilot study. BMJ Open. 2016;6:e009721. 26733570 10.1136/bmjopen-2015-009721 PMC4716232

[108] Qian K, Xu H, Chen Z, Zheng Y. Advances in pulmonary rehabilitation for children with bronchial asthma. Zhejiang Da Xue Xue Bao Yi Xue Ban. 2023;52:518–25. 37643985 10.3724/zdxbyxb-2023-0081 PMC10495252

[109] Agache I, Eguiluz-Gracia I, Cojanu C, Laculiceanu A, Del Giacco S, Zemelka-Wiacek M, et al. Advances and highlights in asthma in 2021. Allergy. 2021;76:3390–407. 34392546 10.1111/all.15054

[110] McGowan CJ, Pyne DB, Thompson KG, Rattray B. Warm-up strategies for sport and exercise: mechanisms and applications. Sports Med. 2015;45:1523–46. 26400696 10.1007/s40279-015-0376-x

[111] Ding L, Luo J, Smith DM, MacKey M, Fu H, Davis M, et al. Effectiveness of warm-up intervention programs to prevent sports injuries among children and adolescents: a systematic review and meta-analysis. Int J Environ Res Public Health. 2022. 10.3390/ijerph19106336. 35627873 10.3390/ijerph19106336 PMC9140806

[112] Grandinetti R, Mussi N, Rossi A, Zambelli G, Masetti M, Giudice A, et al. Exercise-induced bronchoconstriction in children: state of the art from diagnosis to treatment. J Clin Med. 2024;13:4558. 39124824 10.3390/jcm13154558 PMC11312884

[113] Stickland MK, Rowe BH, Spooner CH, Vandermeer B, Dryden DM. Effect of warm-up exercise on exercise-induced bronchoconstriction. Med Sci Sports Exerc. 2012;44:383–91. 21811185 10.1249/MSS.0b013e31822fb73a

[114] Elkins MR, Brannan JD. Warm-up exercise can reduce exercise-induced bronchoconstriction. Br J Sports Med. 2013;47:657–8. 23038787 10.1136/bjsports-2012-091725

[115] Scherr J, Wolfarth B, Christle JW, Pressler A, Wagenpfeil S, Halle M. Associations between Borg’s rating of perceived exertion and physiological measures of exercise intensity. Eur J Appl Physiol. 2013;113:147–55. 22615009 10.1007/s00421-012-2421-x

[116] Arney BE, Glover R, Fusco A, Cortis C, de Koning JJ, van Erp T, et al. Comparison of RPE (rating of perceived exertion) scales for session RPE. Int J Sports Physiol Perform. 2019;14:994–6. 30569764 10.1123/ijspp.2018-0637

[117] Mendes MA, da Silva I, Ramires V, Reichert F, Martins R, Ferreira R, et al. Metabolic equivalent of task (METs) thresholds as an indicator of physical activity intensity. PLoS ONE. 2018;13:e0200701. 30024953 10.1371/journal.pone.0200701 PMC6053180

[118] Ainsworth BE, Haskell WL, Herrmann SD, Meckes N, Bassett DR Jr, Tudor-Locke C, et al. 2011 compendium of physical activities: a second update of codes and MET values. Med Sci Sports Exerc. 2011;43:1575–81. 21681120 10.1249/MSS.0b013e31821ece12

[119] Glass S, Dwyer GB, American College of Sports M. ACSM’S metabolic calculations handbook. Philadelphia: Lippincott Williams & Wilkins; 2007.

[120] Ainsworth BE, Haskell WL, Whitt MC, Irwin ML, Swartz AM, Strath SJ, et al. Compendium of physical activities: an update of activity codes and MET intensities. Med Sci Sports Exerc. 2000;32:S498–504. 10993420 10.1097/00005768-200009001-00009

[121] Ainsworth BE, Haskell WL, Leon AS, Jacobs DR Jr, Montoye HJ, Sallis JF, et al. Compendium of physical activities: classification of energy costs of human physical activities. Med Sci Sports Exerc. 1993;25:71–80. 8292105 10.1249/00005768-199301000-00011

[122] Thompson L, Charitos S, Bird J, Marshall P, Brigden A. Exploring the use of smartwatches and activity trackers for health-related purposes for children aged 5 to 11 years: systematic review. J Med Internet Res. 2025;27:e62944. 39870369 10.2196/62944 PMC11811667

[123] Oliveira TRA, Fernandes ATDNSF, Santino TA, Menescal FEPDS, Nogueira PAMS. Effects of using wearable devices to monitoring physical activity in pulmonary rehabilitation programs for chronic respiratory diseases: a systematic review protocol. PLoS ONE. 2024;19:e0308109. 39058745 10.1371/journal.pone.0308109 PMC11280527

[124] Greiwe J, Nyenhuis SM. Wearable technology and how this can be implemented into clinical practice. Curr Allergy Asthma Rep. 2020;20:36. 32506184 10.1007/s11882-020-00927-3 PMC7275133

[125] Ghanvatkar S, Kankanhalli A, Rajan V. User models for personalized physical activity interventions: scoping review. JMIR Mhealth Uhealth. 2019;7:e11098. 30664474 10.2196/11098 PMC6352015

[126] Lang L, Ma M, Zhao H, Zhang J, Liu S, Liu H. Global research trends in obesity-related asthma (2004–2023): a bibliometric analysis. Front Nutr. 2025;12:1528366. 40248034 10.3389/fnut.2025.1528366 PMC12003137

[127] Deschildre A, Pin I, El Abd K, Belmin-Larrar S, El Mourad S, Thumerelle C, et al. Asthma control assessment in a pediatric population: comparison between GINA/NAEPP guidelines, childhood asthma control test (C-ACT), and physician’s rating. Allergy. 2014;69:784–90. 24725204 10.1111/all.12402

[128] Chu F, Kappel N, Akel M, Press VG, Alexander JT, Volerman A. Validity of the childhood asthma control test in diverse populations: a systematic review. Pediatr Pulmonol. 2023;58:1322–36. 36718492 10.1002/ppul.26342 PMC10121871

[129] Koolen BB, Pijnenburg MW, Brackel HJ, Landstra AM, van den Berg NJ, Merkus PJ, et al. Comparing global initiative for asthma (GINA) criteria with the childhood asthma control test (C-ACT) and asthma control test (ACT). Eur Respir J. 2011;38:561–6. 21406508 10.1183/09031936.00173710

[130] Andrade LB, Silva DA, Salgado TL, Figueroa JN, Lucena-Silva N, Britto MC. Comparison of six-minute walk test in children with moderate/severe asthma with reference values for healthy children. J Pediatr. 2014;90:250–7. 10.1016/j.jped.2013.08.006 24184268

[131] de Aguiar KB, Anzolin M, Zhang L. Global prevalence of exercise-induced bronchoconstriction in childhood: a meta-analysis. Pediatr Pulmonol. 2018;53:412–25. 29364581 10.1002/ppul.23951

[132] Klain A, Giovannini M, Pecoraro L, Barni S, Mori F, Liotti L, et al. Exercise-induced bronchoconstriction, allergy and sports in children. Ital J Pediatr. 2024;50:47. 38475842 10.1186/s13052-024-01594-0 PMC10935963

[133] Greiwe J, Cooke A, Nanda A, Epstein SZ, Wasan AN, Shepard KV, et al. Work group report: perspectives in diagnosis and management of exercise-induced bronchoconstriction in athletes. J Allergy Clin Immunol Pract. 2020;8:2542–55. 32636147 10.1016/j.jaip.2020.05.020

[134] Dreßler M, Friedrich T, Lasowski N, Herrmann E, Zielen S, Schulze J. Predictors and reproducibility of exercise-induced bronchoconstriction in cold air. BMC Pulm Med. 2019;19:94. 31097027 10.1186/s12890-019-0845-3 PMC6524332

[135] Alving K. FeNO and the prediction of exercise-induced bronchoconstriction. J Allergy Clin Immunol Pract. 2018;6:863–4. 29747989 10.1016/j.jaip.2017.12.038

[136] Kim K, Cho HJ, Yoon JW, Choi SH, Sheen YH, Han MY, et al. Exhaled nitric oxide and mannitol test to predict exercise-induced bronchoconstriction. Pediatr Int. 2018;60:691–6. 29786927 10.1111/ped.13599

[137] Dreßler M, Salzmann-Manrique E, Zielen S, Schulze J. Exhaled NO as a predictor of exercise-induced asthma in cold air. Nitric Oxide. 2018;76:45–52. 29526567 10.1016/j.niox.2018.03.004

[138] Global Initiative for Asthma (GINA). Global strategy for asthma management and prevention. 2025. https://ginasthma.org/2025-gina-strategy-report/. Accessed May 28 2025.

[139] Vichara-Anont I, Lumkul L, Phinyo P, Wongsa C, Thongngarm T. Efficacy and safety of maintenance regimens for adolescent and adult asthmatics with exercise-induced bronchospasm: systematic review and network meta-analysis. J Allergy Clin Immunol Pract. 2025;13:1755–67.e3. 40021120 10.1016/j.jaip.2025.02.018

[140] Backer V, Mastronarde J. Pharmacologic strategies for exercise-induced bronchospasm with a focus on athletes. Immunol Allergy Clin North Am. 2018;38:231–43. 29631732 10.1016/j.iac.2018.01.011

[141] Dickinson J, Amirav I, Hostrup M. Nonpharmacologic strategies to manage exercise-induced bronchoconstriction. Immunol Allergy Clin North Am. 2018;38:245–58. 29631733 10.1016/j.iac.2018.01.012

[142] Jayasinghe H, Kopsaftis Z, Carson K. Asthma bronchiale and exercise-induced bronchoconstriction. Respiration. 2015;89:505–12. 26068579 10.1159/000433559

[143] Bonini M, Silvers W. Exercise-induced bronchoconstriction: background, prevalence, and sport considerations. Immunol Allergy Clin North Am. 2018;38:205–14. 29631730 10.1016/j.iac.2018.01.007

[144] Wilkinson M, Hart A, Milan SJ, Sugumar K. Vitamins C and E for asthma and exercise-induced bronchoconstriction. Cochrane Database Syst Rev. 2014;2014:CD010749. 10.1002/14651858.CD010749.pub2 PMC6513032 24936673

